# High Salinity and High Temperature Stable Colloidal Silica Nanoparticles with Wettability Alteration Ability for EOR Applications

**DOI:** 10.3390/nano11030707

**Published:** 2021-03-11

**Authors:** Nanji J. Hadia, Yeap Hung Ng, Ludger Paul Stubbs, Ole Torsæter

**Affiliations:** 1 Institute of Chemical and Engineering Sciences, Agency for Science, Technology, and Research (A*STAR), 1 Pesek Road, Jurong Island, Singapore 627833, Singapore; ludger_paul@ices.a-star.edu.sg; 2 Department of Geoscience and Petroleum, Norwegian University of Science and Technology (NTNU), 7031 Trondheim, Norway; ole.torsater@ntnu.no

**Keywords:** enhanced oil recovery, nanotechnology for EOR, nanoparticles stability, reservoir condition

## Abstract

The stability of nanoparticles at reservoir conditions is a key for a successful application of nanofluids for any oilfield operations, e.g., enhanced oil recovery (EOR). It has, however, remained a challenge to stabilize nanoparticles under high salinity and high temperature conditions for longer duration (at least months). In this work, we report surface modification of commercial silica nanoparticles by combination of zwitterionic and hydrophilic silanes to improve its stability under high salinity and high temperature conditions. To evaluate thermal stability, static and accelerated stability analyses methods were employed to predict the long-term thermal stability of the nanoparticles in pH range of 4–7. The contact angle measurements were performed on aged sandstone and carbonate rock surfaces to evaluate the ability of the nanoparticles to alter the wettability of the rock surfaces. The results of static stability analysis showed excellent thermal stability in 3.5% NaCl brine and synthetic seawater (SSW) at 60 °C for 1 month. The accelerated stability analysis predicted that the modified nanoparticles could remain stable for at least 6 months. The results of contact angle measurements on neutral-wet Berea, Bentheimer, and Austin Chalk showed that the modified nanoparticles were able to adsorb on these rock surfaces and altered wettability to water-wet. A larger change in contact angle for carbonate surface than in sandstone surface showed that these particles could be more effective in carbonate reservoirs or reservoirs with high carbonate content and help improve oil recovery.

## 1. Introduction

Stages of oil recovery from petroleum reservoirs typically include primary, secondary, and tertiary recoveries processes. After primary (due to natural pressure energy of the reservoir) and secondary recovery (typically water or gas injection to maintain reservoir pressure) processes become uneconomical, tertiary recovery (also known as enhanced oil recovery (EOR)) methods are employed to improve oil recovery. In EOR, properties of reservoir rock and fluids and their interactions are altered to favorable conditions to mobilize residual oil trapped in oil reservoirs [1]. Two common EOR processes include thermal EOR and chemical EOR methods. In thermal EOR, heat energy is applied to the reservoir, usually by hot water or steam injection, to reduce the viscosity of oil and thus increase its mobility [2]. In chemical EOR methods, chemicals such as alkalis, polymers, and surfactants are injected into reservoirs to create favorable crude oil/brine/rock interactions to increase displacement and sweep efficiencies. Polymers such as hydrolyzed polyacrylamide (HPAM) and xanthan gum are commonly used to increase the viscosity of injection water and thus improve oil/water mobility ratio for effective displacement of oil by water [3]. Surfactants are primarily used to lower the interfacial tension (IFT) between oil and water and thus decrease capillary forces to mobilize trapped oil at pore throats [4].

These chemicals, however, tend to degrade and therefore loose its effectiveness under high temperature and high salinity conditions usually present in oil reservoirs. Moreover, a significant portion of the injected chemicals adsorb on the rock surfaces which leads to loss of these chemicals and eventually increases the project costs. These specially designed formulations of polymers and surfactants for harsh reservoir conditions are costly and make chemical EOR projects economically less viable. One of the ways to reduce the cost associated with chemical EOR is to replace or combine expensive with cheap and yet effective materials or chemicals. In this regard, nanotechnology may prove to be promising due to rapid progress and advancements in novel nanomaterials for various industries ranging from biomedical to personal care to oil and gas.

In recent years, nanotechnology is gaining increasing attention in oil and gas industries. Many research articles have provided a comprehensive overview on potential applications of nanotechnology in this area [5,6,7,8,9]. For EOR applications, at laboratory scale, nanoparticles were studied as performance improvers for polymer [10,11] and surfactant [12,13] flooding processes, wettability modifiers [14,15,16,17,18], emulsion stabilizers [19,20,21,22], etc. Among various types of nanoparticles, silica nanoparticles (SNP) are extensively studied for EOR applications. For a successful EOR application, a long-term colloidal stability of nanoparticles in harsh reservoir conditions is very crucial. The nanoparticle dispersions tend to lose colloidal stability under high salinity and high temperature conditions due to van der Waals forces and formation of aggregates over time. The stability of nanodispersions can be improved by electrostatic or steric stabilization mechanisms [23] to make them suitable for EOR applications. Recently, (3-glycidyloxypropyl) trimethoxysilane (GLYMO), a hydrophilic silane, has been proposed by many researchers to stabilize nanodispersions in hostile environments [24,25,26,27]. Worthen et al. [24] compared silica nanoparticles of 7–20 nm size stabilized by three types of nonionic ligands namely, GLYMO, polyethylene glycol (PEG), and zwitterionic sulfobetaine (SB) in seawater and American Petroleum Institute (API) brine. Their results showed that GLYMO and SB ligands were able to stabilize nanodispersions up to 80 °C for over 30 days in pH 3.5 API brine. The stability at pH > 3.5 is not reported. GLYMO also provided colloidal stability for 3 days even at 120 °C. Jang et al. [25] achieved colloidal stability of silica nanofluid up to salinity of 20% at 90 °C by modifying silica nanoparticles with GLYMO. Their wettability tests on oil-wet carbonate rocks showed effective wettability alteration to neutral- and water-wet by modified silica nanofluids. Griffith and Daigle [26,27] have shown that GLYMO-modified silica nanoparticles helped stabilize oil-in-water Pickering emulsions.

Wettability of reservoir rocks is a crucial parameter that controls oil production and affects the oil recovery during waterflooding. Oil recovery from oil-wet reservoirs, e.g., carbonates, is generally lower than intermediate- or water-wet reservoirs [17,28]. In water-wet reservoirs, at a given oil saturation, the oil occupies larger pores and water relative permeability is lower, which results in higher oil recovery. In oil-wet reservoirs, the water breaks through early and makes waterflooding uneconomical due to increased water-cut after breakthrough. Wettability also affects the oil recovery from heterogeneous reservoirs with zones of high and low permeability. In the case of water-wet rocks, water easily imbibes into low permeability zones, thus less bypassing occurs and oil recovery increases. In oil-wet rocks, water cannot spontaneously imbibe into low permeability zones due to high capillary forces, thus bypassing low permeability zones and hence lower oil recovery. To improve oil recovery from such oil-wet reservoirs, it is necessary to alter the wettability of rock surfaces to intermediate- or water-wet. Surfactants are traditionally used as wettability modifying agents for oil-wet reservoirs. Different types of surfactants have been studied to alter the wettability of oil-wet reservoirs [29,30,31,32]. During surfactant flooding, the surfactant molecules adsorb on the rock surface by various mechanisms and render it water-wet. The mechanism of adsorption depends on the type of surfactant, e.g., anionic, cationic, or non-ionic [33].

Recently, nanoparticles have been extensively studied for their potential to alter the wettability of rock surfaces. Xu et al. [14] formulated an ultra-low interfacial tension (IFT) nanofluid consisting of surfactant and silica nanoparticles for very low permeability (0.2–0.3 mD) reservoirs. Their wettability experiments demonstrated that the silica nanoparticles could effectively alter the wettability of the rock, making it become more water-wet with increasing silica nanoparticle concentration. Bayat et al. [15] used aluminum oxide (Al_2_O_3_), titanium dioxide (TiO_2_), and silicon dioxide (SiO_2_) nanoparticles to study the adsorption and the resulting wettability alteration of limestone surface at different temperatures. The results showed wettability change of intermediate-wet limestone to water-wet due to adsorption of nanoparticles on the surface. Maghzi et al. [16] performed experiments to study the effect of adding silica nanoparticles to a polymer solution on heavy oil recovery in a five-spot glass micromodel. Based on oil recovery and contact angle measurements, they concluded that adding silica nanoparticles to the polymer solution changed the glass surface from oil-wet to water-wet and resulted in 10% higher oil recovery than by polymer flooding only. Al-Anssari et al. [17] also found that silica nanoparticles have the ability to alter the wettability of calcite surfaces from oil-wet to strongly water-wet by irreversible adsorption. Recently, Kanj et al. [18] developed a carbon nanofluid system for EOR application in high temperature and high salinity carbonate reservoirs. Their contact angle experiments showed effective wettability alteration of carbonate rock surfaces from oil-wet to water-wet.

In this study, we report functionalization and surface modification of commercial colloidal silica nanoparticles for high salinity and high temperature conditions. We compared the long-term stability of GLYMO-, SBS-, and GLYMO-SBS-modified nanodispersions at high temperature by static and accelerated stability analyses methods. The accelerated stability analysis simulated long-term stability in shorter period by subjecting the samples to centrifugal force and therefore predict the stability duration. Finally, contact angle measurements were performed in high salinity conditions on neutral-wet sandstone and carbonate rock surfaces to wettability alteration capability of the nanoparticles and therefore its suitability for EOR applications. The results showed that the GLYMO-SBS modified nanodispersion exhibited excellent stability in high salinity brines at 60 °C for at least 6 months and altered the wettability of neutral-wet sandstone and carbonate rocks to water-wet.

## 2. Experimental

### 2.1. Materials

Levasil OF50, an aqueous dispersion of colloidal silica (solid content 15 wt%, specific surface area 500 m^2^/g, pH 10) was supplied by Nouryon Asia Pte Ltd., Singapore. The particles were used as received for further modification. The average DLS (dynamic light scattering) size of the particles was about 22 nm. Moreover, [3-(*N,N*-Dimethylamino)propyl]trimethoxysilane (>96.0%(GC), TCI), 1,3-propanesultone (≥99%, New York, NY, USA, Sigma-Aldrich), acetone (99.8%, Extra Dry, Acros Organics, Geel, Belgium), acetic acid (glacial, ≥99.8%, Sigma-Aldrich, New York, NY, USA), and (3-glycidyloxypropyl)trimethoxysilane (GLYMO, ≥98%, Sigma-Aldrich, New York, NY, USA) were used as received.

Two types of brines, synthetic seawater (SSW) and 3.5% NaCl, were prepared by dissolving different salts in ultrapure water followed by vacuum filtration using 0.22 µ filter. The composition of SSW is given in Table 1. Berea, Bentheimer, and Austin Chalk rock samples (Kocurek Industries, Caldwell, TX, USA) of dimensions 20 × 10 × 5 mm^3^ were used for contact angle measurements. The mineral composition of the rock samples measured by X-ray diffraction (XRD) is shown in Table 2. A stock tank light crude oil (density: 0.852 g/cc; viscosity: 2.43 mPa·s at room temperature) was used for saturation and aging of rock samples and contact angle measurements. The saturates, aromatics, resins, and asphaltenes (SARA) composition of the crude oil was 38.5%, 56.1%, 5.3%, and 0.1%, respectively.

**Table 1:** Composition of synthetic seawater.

Salts	Concentration (g/L)	Salts	Concentration (g/L)
NaCl	27.03	MgCl_2_·6H_2_O	11.23
CaCl_2_·2H_2_O	1.76	Na_2_SO_4_	4.81

**Table 2:** Mineral composition of rock samples by XRD.

Mineral, %	Berea	Bentheimer [34]	Austin Chalk
Quartz	90.87	99.0	–
Albite	0.89	–	–
Sanidine	0.46	–	–
Muscovite	3.75	–	–
Kaolinite	1.92	0.7	–
Rutile	–	0.3	–
Clinochlore	0.97	–	–
Dolomite	1.13	–	–
Calcite	–	–	100

### 2.2. Methods

#### 2.2.1. Synthesis of 3-(Dimethyl(3-(Trimethoxysilyl)Propyl)-Ammonio)Propane-1-Sulfonate (SBS)

The synthesis of zwitterionic silane, SBS, was adapted from Estephan et al. [35]. In a 100-mL Schlenk flask, 8 g of [3-(*N,N*-dimethylamino)propyl]trimethoxysilane was diluted with 40 mL of anhydrous acetone under argon. Then, 3.4 mL of 1,3-propane sultone was subsequently added using an argon-purged syringe. The reaction mixture was stirred vigorously for 6 h. White precipitate was then collected through filtration, and then washed with acetone for three times. The collected solid was immediately vacuum dried for 24 h and stored under argon before use.

#### 2.2.2. Surface Functionalization of Silica Nanoparticles

**Figure 1:**
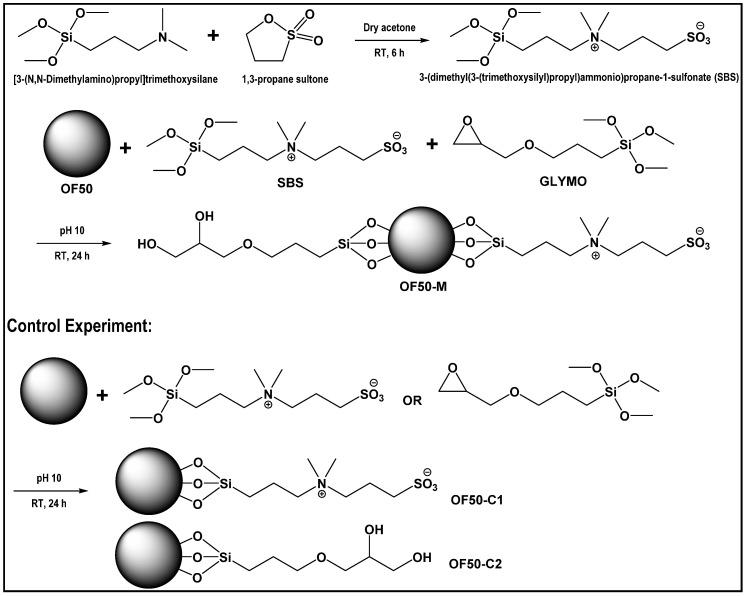
Synthesis of 3-(dimethyl(3-(trimethoxysilyl)propyl)-ammonio)propane-1-sulfonate (SBS) and surface modification of Levasil OF50 using SBS and (3-glycidyloxypropyl) trimethoxysilane (GLYMO).

For synthesis of surface-functionalized colloidal silica referred as OF50-M, as illustrated in Figure 1, 8 g of OF50 was added to a 20-mL vial. SBS (150 mg, 0.46 mmol) and GLYMO (150 µL, 0.68 mmol) were added to the colloidal dispersion under vigorous stirring. After the opaque liquid turned transparent, the mixture was stirred at 200 rpm for 24 h at room temperature. The modified silica nanodispersions were then diluted to 1 wt.% using 3.8% SSW or 3.5% NaCl, and the pH was adjusted to about 4 using glacial acetic acid.

Silica nanoparticles in the form of dry powder were prepared for chemical analysis. Pristine OF50 (OF50-UM) was diluted to ca. 5 wt.% with ultrapure water and centrifuged at 14,000 rpm (Allegra^TM^ 25R Centrifuge Beckman Coulter) for 2 h. The collected solid was washed twice with water, rinsed with methanol once, and dried under vacuum at room temperature for 2 days. OF50-M was dialyzed in DI water for 1 week using a dialysis membrane (Spectra/Pro^®^7 MWCO 50 kD). The purified sample was subjected to similar centrifugation and washing procedure for attaining the pure solid. Similar to the modification protocol for OF50-M, two control samples, OF50-C1 and OF50-C2, were also prepared by functionalizing solely with SBS and GLYMO, respectively.

#### 2.2.3. Structural and Property Characterization

The Fourier-transform infrared (FTIR) spectra for pristine and functionalized silica were obtained from a PerkinElmer Frontier^TM^ FT-IR/NIR spectrometer with KBr as background. Particles size analysis of colloidal samples (concentration 1 wt.%) was done by dynamic light scattering (DLS) using Malvern Zetasizer Nano ZS. Particle morphology was observed using JEOL JSM7900F field emission scanning electron microscope (FE-SEM) at accelerating voltage of 1.5 kV under GBSH mode.

#### 2.2.4. Stability Analysis of Nanoparticle Dispersions

The thermal and long-term stability tests of nanodispersions were performed (i) by visual observations, (ii) by turbidity scanning test using Turbiscan^TM^ instrument for real-time monitoring of transmission intensities over time, and (iii) using accelerated stability analysis. In turbidity scanning tests, the transmission profiles were recorded at every 3 h for 7 days. For this purpose, 1 wt.% OF50-M in SSW and 3.5% NaCl brines at pH 6 were subjected to turbidity scanning tests.

The accelerated stability analysis helps simulate long-term stability in shorter period of time by subjecting the samples to centrifugal force. A LUMiSizer instrument (LUM GmbH, Germany) was used for this purpose. In this study, the stability duration of 6 month was simulated in the runtime of about 31 h and transmission signals were recorded by a SEPView software [36] provided with the instrument. All accelerated stability analysis measurements were performed at 60 °C for 1 wt.% nanoparticles in SSW and 3.5% NaCl brine in the pH range of 4–7. For OF50 and OF50-C1, samples at pH 4 and 7 were used for accelerated stability analysis. All the samples were run in duplicates to counter-balance the weight on the rotor of the instrument. The size of nanoparticles was analyzed at room temperature for fresh samples as well as samples that were subjected to turbidity scan and accelerated stability analysis.

#### 2.2.5. Contact Angle Measurements

**Figure 2:**
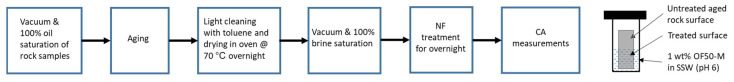
Procedure to prepare rock samples for contact angle measurements.

In these experiments, the rock samples of dimensions 20 × 20 × 5 mm^3^ were first cut into two equal parts and washed thoroughly with ethanol, blow-dried, and placed in an oven at 70 °C overnight for complete drying. Figure 2 depicts the procedure followed for rock sample preparation for contact angle measurements. The dried chips were then saturated with the crude oil using vacuum saturation. The rock samples with 100% oil saturation were placed in an oven at 70 °C for aging to obtain representative wettability conditions. Berea and Bentheimer Sandstone rock samples were aged for 14 days, whereas Austin Chalk rock samples were aged for 2 days. At the end of aging period, all the rock samples were lightly rinsed with toluene to remove any free crude oil yet preserving the original wettability condition of the pore surfaces. The rock samples were then dried overnight in the oven at 70 °C. The dried rock chips were subsequently saturated with brines using vacuum saturation and used for contact angle measurements. To observe the wettability alteration by nanoparticles on the same rock sample, about half the rock sample was treated with nanofluids by immersing in the nanofluid for overnight and the other half was left untreated. It was thus possible to measure the contact angles on untreated and treated part of the rock chips without using duplicate rock chips. 

Contact angle measurements were performed at room temperature by captive bubble method using an optical contact angle meter (Model: OCA50, DataPhysics Instruments GmbH). For each measurement, a few hours were allowed for equilibration; all the measurement values reported herein were equilibrium values.

## 3. Results and Discussion

### 3.1. Nanoparticle Modification and Characterization

**Figure 3:**
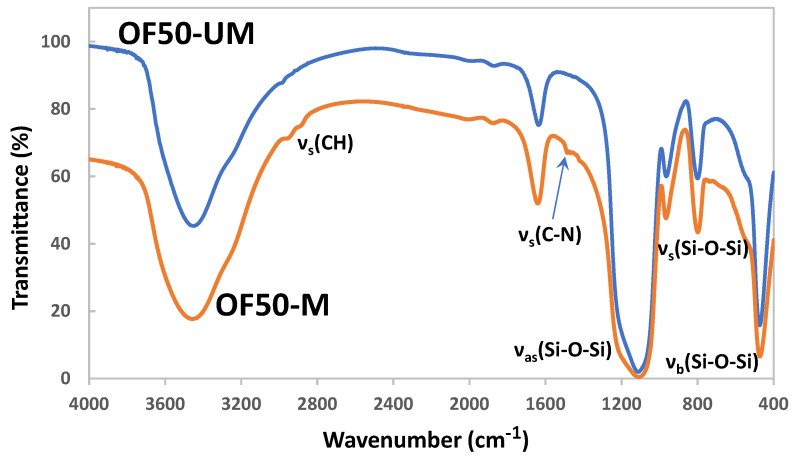
FTIR spectra of pristine OF50 (OF50-UM) and surface-modified/functionalized colloidal silica (OF50-M).

**Figure 4:**
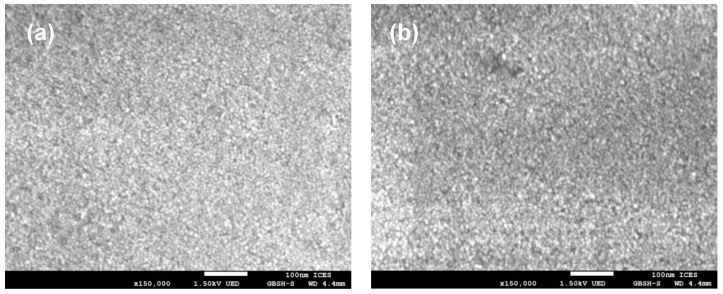
Scanning electron micrographs of (**a**) pristine OF50 and (**b**) OF50-M.

The FTIR spectra of pristine and functionalized silica particles are shown in Figure 3. For the pristine unmodified OF50-UM, strong absorption peaks at 469, 807, and 1100 cm^−1^ correspond to Si-O-Si bending (ν_b_), the Si-O-Si symmetric (ν_s_), and asymmetric (ν_as_) stretching vibrations, respectively. For SBS and GLYMO functionalized OF50-M, small intensity peaks between 3000 and 2800 cm^−1^ are attributed to the stretching vibrations of the methylene groups from GLYMO, which confirms the successful surface chemical modification of OF50. For the identification of sulfobetaine functionality, the absorption peak at 1485 cm^−1^ corresponding to the C-N stretch from -N^+^-(CH_3_)_2_ is used. The peaks arising from ca. 1220 cm^−1^ ν_as_(SO_3_^−^) and 1040 cm^−1^ ν_s_(SO_3_^−^) are not identified due to overlapping with strong Si-O-Si peak. Figure 4 shows SEM images of pristine OF50 and OF50-M nanoparticles. It can be observed that the size of the nanoparticles did not change significantly after the modification.

### 3.2. Nanoparticle Dispersion Stability

**Figure 5:**
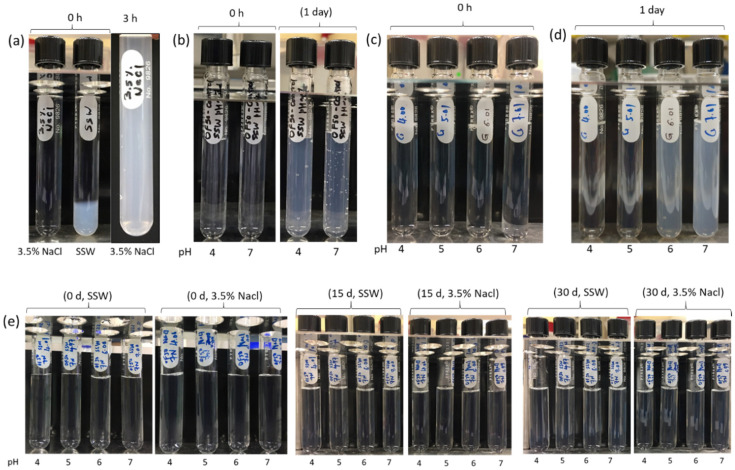
Thermal stability of 1 wt.% (**a**) OF50-UM in synthetic seawater (SSW) and 3.5% NaCl, (**b**) SBS modified particles (OF50-C1) in SSW at pH 4 and 7, (**c**) nanoparticles modified with GLYMO (OF50-C2) in SSW at pH 4–7 at 0 h, (**d**) OF50-C2 in SSW at pH 4–7 after 1 day, and (**e**) OF50-M in SSW and 3.5% NaCl brine at pH 4–7 for 30 days at 60 °C.

The thermal stability of unmodified, control, and modified nanoparticles dispersions was monitored at 60 °C for 30 days, and pictures were taken at regular intervals for visual observations. Figure 5 shows the photographs of the nanoparticle dispersions. It can be observed that OF50—UM nanoparticles precipitated out almost instantaneously in SSW, and after 3 h in 3.5% NaCl (Figure 4a). This shows that dispersions of the particles in their native form are not stable in high salinity conditions. The SBS-modified particles (OF50-C1) at pH 4 and 7 remained stable a little longer than unmodified nanoparticles but aggregated within 24 h, as can be observed in Figure 5b. Nanoparticles modified with GLYMO (OF50-C2) at pH 6 and 7 also aggregated within a day as can be observed in Figure 5d. These results show that only SBS or GLYMO modification is not enough to stabilize these nanodispersions in high salinity and high temperature conditions. On the other hand, dispersions of OF50-M nanoparticles showed excellent thermal stability in 3.5% NaCl as well as SSW in the pH range of 4–7 for 30 days without any sign of aggregation (Figure 5e). Modification of OF50 nanoparticles with combination of SBS and GLYMO helped to stabilize the dispersions for very long time. We anticipate that the optimum balance of surface coverage of hydroxyl and sulfobetaine functional groups played the pivotal role for ensuring the long-term saline stability of OF50-M nanodispersions. While the SBS-covered OF50-C1 was still susceptible to salt-induced destabilization due to presence of unmodified surface silanol group (-Si-OH), presence of grafted GLYMO was able to further enhance the solvation of the nanoparticles, which promoted the saline stability of dispersions at elevated temperature. 

**Figure 6:**
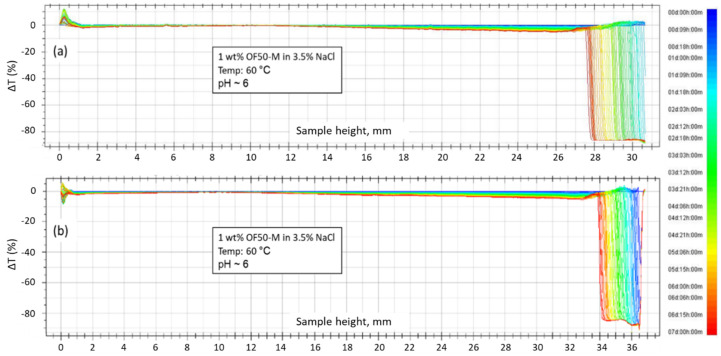
Transmission (ΔT) profiles obtained by Turbiscan^TM^ at 60 °C for 1 wt.% OF50-M nanoparticles in (**a**) SSW and (**b**) 3.5% NaCl brine for 1-week duration.

Results of a real-time turbidity monitoring of 1 wt.% OF50-M in SSW and 3.5% NaCl brine at pH 6 by Turbiscan^TM^ are shown in Figure 6. The results are presented in the form of transmission signals vs. height (0 mm represents bottom of the sample) of the sample for 7 days with each profile recorded every 3 h. No significant change in the transmission (ΔT) intensity was observed for 7 days across the height of the sample. This indicated that the particles were not aggregated and the dispersions remained stable over the duration of the test. The right side of the curves shows a shift of transmission profiles towards left (blue to red). This may be attributed to evaporation of the water in the sample vials as they were not completely filled with the nanofluid.

**Figure 7:**
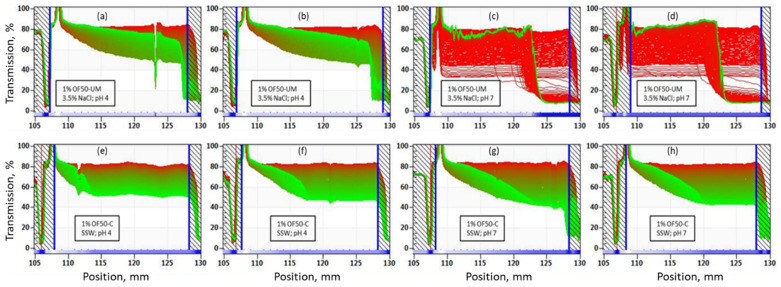
Accelerated stability analysis at 60 °C for 1 wt.% (**a**,**b**) OF50-UM in 3.5% NaCl brine at pH 4, (**c**,**d**) OF50-UM in 3.5% NaCl brine at pH 7, (**e**,**f**) OF50-C1 in SSW at pH 4, and (**g**,**h**) OF50-C1 in SSW at pH 7. The simulated stability duration was 6 months and runtime of experiment was 31 h.

**Figure 8:**
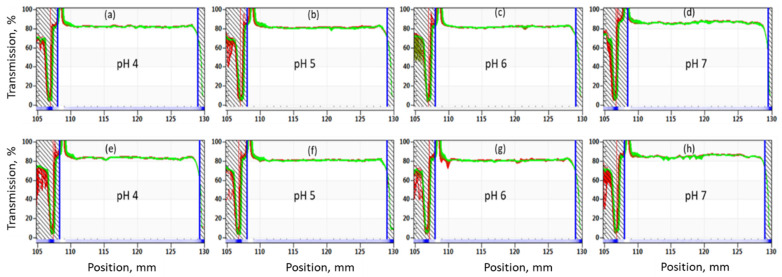
Accelerated stability analysis at 60 °C for 1 wt.% OF50-M in 3.5% NaCl brine at (**a**,**e**) pH 4, (**b**,**f**) pH 5, (**c**,**g**) pH 6, and (**d**,**h**) pH 7. The simulated stability duration was 6 months and runtime of experiment was 31 h. Samples were run in duplicate.

**Figure 9:**
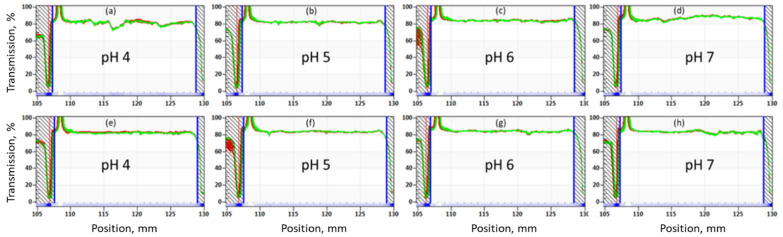
Accelerated stability analysis at 60 °C for 1 wt.% OF50-M in SSW at (**a**,**e**) pH 4, (**b**,**f**) pH 5, (**c**,**g**) pH 6, and (**d**,**h**) pH 7. The simulated stability duration was 6 months and runtime of experiment was 31 h. Samples were run in duplicate.

Results of accelerated stability analysis of OF50-UM, OF50-C1, and OF50-M nanofluids are shown in Figure 7, Figure 8 and Figure 9 (with one repeat measurement for each nanofluid). OF50—C2 was not further tested due to insufficient colloidal stability. The time of measurement is indicated by color of the curves, from beginning (red) to end (green). The x-axis represents the height of the sample from top (130 mm) to bottom (105 mm). From Figure 7a–d, it can be observed that light transmission decreased with time from initial 80% to about 50% for nanofluid dispersions with 1 wt.% OF50-UM nanoparticles in 3.5% NaCl brine at pH 4 and 7. The decreased light transmission indicated aggregation of nanoparticles with time. For OF50-UM in 3.5% NaCl brine at pH 7, a sudden reduction in transmission from 80% to 5% occurred at about 124 mm (about 6 mm from bottom of the sample vial) as can be observed from Figure 7c,d. The formation of gel started after about 48 min of the test that is equivalent to simulated time of about 3 days. A significant reduction in transmission clearly showed a formation of gel and reduced the light transmission.

From Figure 7e–h, it can be observed that the nanofluids with 1% OF50-C1 also showed a decrease in % transmission from about 80% to 50% at pH 4 and to 40% at pH 7 during the runtime of 31 h. This also clearly shows that the surface modification with zwitterionic silane (SBS) only is not sufficient to stabilize the nanodispersions.

The transmission profiles of 1 wt.% OF50-M in 3.5% NaCl and SSW at different pH are shown in Figure 8 and Figure 9. It can be observed that the transmission intensity did not vary significantly across the height of all the samples for experiment time of 31 h (equivalent to simulated stability time of 6 months). These results clearly indicate that 1wt% OF50-M nanoparticles remain stable for at least 6 months at 60 °C in both types of brines. This can be attributed to the synergy of SBS and GLYMO in stabilizing the nanoparticles against aggregation, as compared to nanoparticles with modification by SBS or GLYMO only.

The results of DLS measurements for OF50-M are shown in Table 3. It can be observed that the sizes of the nanoparticles were nearly the same in SSW and 3.5% NaCl brine. This indicates that the colloidal stability of modified nanoparticles was not affected by the presence of divalent ions in SSW. This combined effect of steric and electrosteric stabilizations imposed by GLYMO and SBS, respectively, is the key factor for enhanced stability of silica nanodispersions stability in high salinity brines. The particle size of samples that were subjected to turbidity scan (for 7 days) and accelerated stability analysis at 60 °C only marginally increased at pH 6 and 7, which further validates good stability of OF50-M particles in high salinity brine at high temperature.

The results of static and accelerated stability analysis and DLS measurements for fresh and treated samples (i.e., subjected static and accelerated stability tests) confirmed excellent stability of OF50-M nanodispersion in SSW and 3.5% NaCl brine at 60 °C. As mentioned earlier, the resulted stability can be attributed to the synergistic effects of GLYMO and SBS and optimum surface coverage of nanoparticles by GLYMO and SBS [35,37].

### 3.3. Wettability Alteration

**Figure 10:**
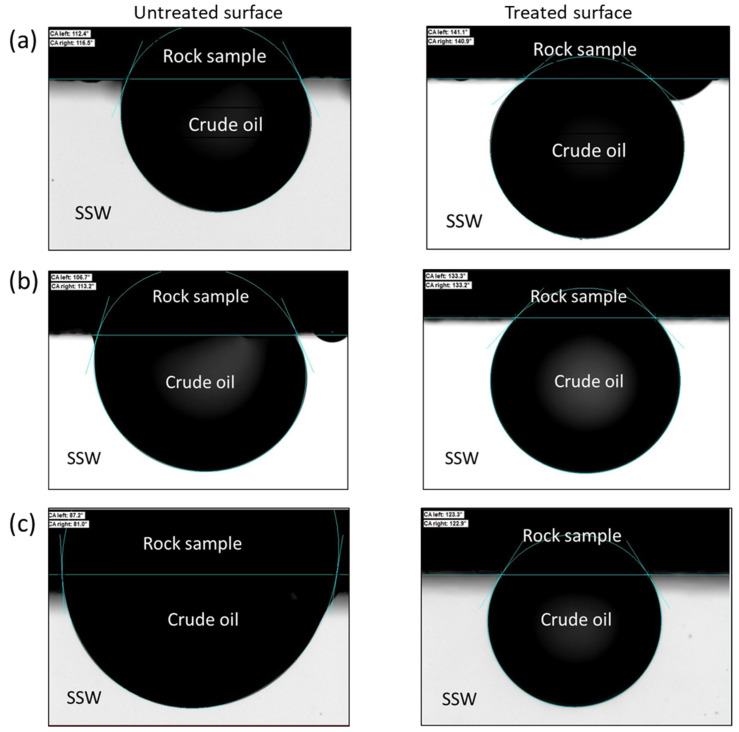
Contact angle measurements on untreated and nanofluid (1 wt.% OF50-M) treated surfaces of (**a**) Bentheimer Sandstone, (**b**) Berea Sandstone, and (**c**) Austin Chalk rock samples at room temperature.

Herein, the results of contact angle measurements are reported and discussed. As mentioned earlier, contact angles were measured on Berea Sandstone, Bentheimer Sandstone, and Austin Chalk rock samples. Figure 10 shows a comparison of contact angles measured on untreated and OF50-M treated sections of the rock chips. The values of contact angles are provided in Table 4. It should be noted that the contact angles are measured through the oil phase.

It is well known that outcrop rocks such as the ones used in this study are naturally strongly water-wet. When aged in crude oil, the polar component from the crude oil adsorbs on the pore walls and change the water-wet rocks to neutral- or oil-wet depending on the crude oil composition and aging time. From Figure 10, it can be observed that Bentheimer and Berea Sandstone samples attained a weakly water-wet condition, whereas neutral-wet condition was observed for Austin Chalk sample after aging in crude oil. When part of these rock samples treated with 1 wt.% OF50-M, the wettability of the surfaces changed to water-wet for all the rock samples. This wettability alteration can be attributed to the adsorption of nanoparticles on the rock surfaces. Due to overall hydrophilic nature of the particles, the surfaces exhibit hydrophilic (water-wet) behavior after the treatment with nanofluid. The change in contact angles for Bentheimer, Berea, and Austin Chalk samples was found to be 28°, 21°, and 39°, respectively (Table 3). Sandstone pore surfaces are negatively charged, whereas those for carbonates are positively charged. Based on the contact angle measurements, it can be concluded that OF50-M nanoparticles have stronger interactions with the carbonate surface and hence more particles may have adsorbed than on the sandstone surfaces. 

These observations show that the modified nanoparticles, OF50-M, have an ability to adsorb on various types of rock surfaces and alter the wettability from neutral- and oil-wet to water-wet conditions, which is an important criterion for more oil recovery from oil-wet petroleum formations.

## 4. Conclusions

Commercial silica nanoparticles were successfully surface modified to improve the colloidal stability of their dispersions under high salinity and high temperature conditions. Static (visual and turbidity) and accelerated stability analyses were performed at high temperatures to predict the long-term thermal stability. The stability tests of unmodified, control, and modified nanodispersions showed that only silane or only GLYMO modification is not sufficient to stabilize these nanofluids, in particular, at pH 6 and 7 and surface modification with combined SBS and GLYMO is necessary. The results of static stability analysis by Turbiscan^TM^ and visual method showed excellent thermal stability in 3.5% NaCl brine and SSW at 60 °C for a week and month, respectively. The accelerated stability analysis predicted that the modified nanoparticles could remain stable at least up to 6 months in the pH range of 4–7. The results of contact angle measurements showed that the modified nanoparticles were able to adsorb on neutral-wet rock surfaces and alter wettability to water-wet. A larger change in contact angle for carbonate surface than in sandstone surface showed that these particles could be more effective in carbonate reservoirs or reservoirs with high carbonate content and help improve oil recovery.

## Figures and Tables

**Table 3:** Dynamic light scattering (DLS) size measurements of 1 wt% OF50-M in synthetic seawater (SSW) and 3.5% NaCl brine when fresh and after turbidity scan and accelerated stability analysis measurements.

pH	Particle Size, nm					
	Fresh Sample		Treated Sample *			
	SSW	3.5% NaCl	SSW-Turbi ^+^	SSW-ASA ^#^	3.5% NaCl-Turbi ^+^	3.5% NaCl-ASA ^#^
4	22.3 ± 0.1	21.6 ± 0.2	–	22.3 ± 0.1	–	21.6 ± 0.1
5	22.5 ± 0.2	21.7 ± 0.1	–	22.7 ± 0.1	–	21.9 ± 0.1
6	22.5 ± 0.2	21.7 ± 0.1	24.2 ± 0.1	24.0 ± 0.1	23.5 ± 0.2	22.9 ± 0.1
7	22.7 ± 0.1	21.8 ± 0.1	–	24.8 ± 0.1	–	22.9 ± 0.1

* Samples first used for turbidity scan and accelerated stability. ^+^ Sample after turbidity scan. ^#^ Samples after accelerated stability analysis. – Samples were not subjected to turbidity scan.

**Table 4:** Contact angles measured on untreated and treated rock surfaces.

Rock Type	Contact Angle *	
	Untreated Surface	Nanofluid Treated Surface
Bentheimer	113°	141°
Berea	110°	131°
Austin Chalk	84°	123°

* mean contact angle.
